# The Joint Action and Learning Initiative: Towards a Global Agreement on National and Global Responsibilities for Health

**DOI:** 10.1371/journal.pmed.1001031

**Published:** 2011-05-10

**Authors:** Lawrence O. Gostin, Eric A. Friedman, Gorik Ooms, Thomas Gebauer, Narendra Gupta, Devi Sridhar, Wang Chenguang, John-Arne Røttingen, David Sanders

**Affiliations:** 1 O'Neill Institute for National and Global Health Law, Georgetown University Law Center, Washington, D.C., United States of America; 2 Institute of Tropical Medicine, Antwerp, Belgium; 3 Medico International, Frankfurt, Germany; 4 Prayas, Chittorgarh, India; 5 Oxford University, Oxford, United Kingdom; 6 Tsinghua University Law School, Beijing, China; 7 Norwegian Knowledge Centre for the Health Services, Oslo, Norway and Institute of Health and Society, University of Oslo, Oslo, Norway; 8 School of Public Health, University of the Western Cape, Bellville, South Africa

## Abstract

Lawrence Gostin and colleagues discuss their work on the Joint Action and Learning Initiative on National and Global Responsibilities for Health (JALI), which aims to secure a global health agreement (such as a Framework Convention on Global Health) that would inform post-Millennium Development Goal global health commitments.

**Summary Points**

- A coalition of civil society organizations and academics are initiating a Joint Action and Learning Initiative on National and Global Responsibilities for Health (JALI) to research key conceptual questions involving health rights and responsibilities, with the goal of securing a global health agreement and supporting civil society mobilization around the human right to health.
- This agreement—such as a Framework Convention on Global Health—would inform post-Millennium Development Goal (MDG) global health commitments.
- Using broad partnerships and an inclusive consultation process, JALI seeks to clarify the health services to which everyone is entitled under the right to health, the national and global responsibilities for securing this right, and global governance structures that can realize these responsibilities and close major health inequities.
- Mutual benefits to countries in the Global South and North would come from a global health agreement that defines national and global health responsibilities.
- JALI aims to respond to growing demands for accountability, and to create the political space that could make a global health agreement possible.

## Introduction

A decade into the 21st century, billions of people have yet to benefit from the health advances of the 20th century. Life expectancy at birth in sub-Saharan Africa is 53 years [1]—only two years higher than in the United States a century ago [2], and 27 years lower than in high-income countries today [1]. The most basic human needs continue to elude the world's poorest people. In 2010, approximately 925 million people were suffering from chronic hunger [3], 884 million people lacked access to clean water, and 2.6 billion people were without access to proper sanitation facilities [4].

Such global health disparities will likely persist until there is fair and effective global governance for health—the organization of national and global norms, institutions, and processes that collectively shape the health of the world's population. Global governance for health goes beyond the health sector. It requires remediating the currently unfair and detrimental health impacts of international regimes (e.g., trade, intellectual property, and finance), and developing stable, responsive, democratic political institutions.

A coalition of civil society and academics, with a shared vision of the “right of everyone to the enjoyment of the highest attainable standard of physical and mental health” [5] (“right to health”), is therefore launching the Joint Action and Learning Initiative on National and Global Responsibilities for Health (JALI). JALI seeks to develop a post–Millennium Development Goal (MDG) framework for global health, one rooted in the right to health and aimed at securing universal health coverage for all people. We seek to clarify the health goods and services to which all people are entitled, national and global responsibilities to secure the health of the world's population, and governance structures required to realize these responsibilities. Our goal is a global agreement, such as a Framework Convention on Global Health, which sets priorities, clarifies national and international responsibilities, ensures accountability, and develops corresponding institutions, such as a Global Health Fund [6],[7].

## Partnerships with Civil Society Organizations

JALI will draw inspiration from, and collaborate with, civil society movements, which are central to securing and ensuring adherence to a global health agreement. Such movements have spurred momentous transformations in health. Advocates changed the world's response to AIDS from one marked by discrimination to one focused on empowering marginalized people and scaling up HIV services. The Campaign to Ban Landmines drove a process that culminated in a treaty banning this indiscriminate weapon.

Civil society campaigns for the right to health, such as those through the People's Health Movement, are already underway [8]. Nongovernmental organizations (NGOs) from the South and North launched a Declaration of Solidarity for a Unified Movement for the Right to Health [9]. JALI is developing the partnerships required to undertake an inclusive process involving research, analysis, and extensive online and regional consultations to gain insight into and build consensus around answers to four foundational questions, and to stimulate coordinated action to reduce health inequities. This bottom-up, research-focused process will develop a detailed understanding of health rights and state obligations, clear targets and benchmarks for success, and effective monitoring and accountability mechanisms. These will add precision to and enhance the effectiveness of international human rights law, which could in turn enhance civil society efforts to hold their own governments to account. By drawing on the voices of civil society and disadvantaged communities, JALI could have the legitimacy and the political support to transform global governance for health.

## Four Defining Questions in Global Health

The four defining questions, and preliminary directions on answers, are:

*1. What are the services and goods guaranteed to every person under the human right to health?*

The World Health Organization (WHO) has placed universal health coverage high on the global health agenda [10], defining three dimensions of coverage: 1) the proportion of the population served; 2) the level of services; and 3) the proportion of health costs covered by prepaid pooled funds [11]. WHO has defined universal coverage “as access to key promotive, preventive, curative and rehabilitative health interventions for all at an affordable cost” [12].

**Figure 1:**
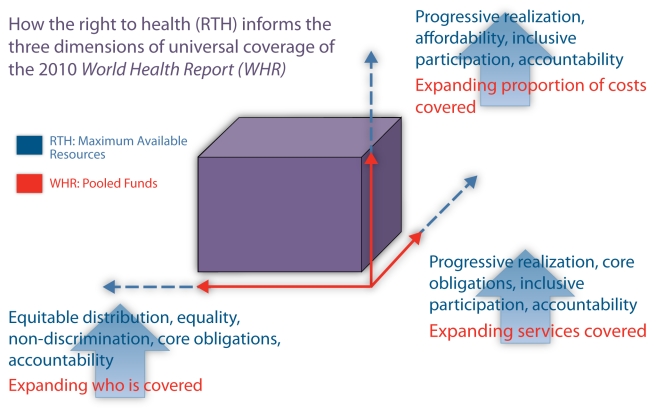
Universal health coverage and the right to health. The pooled funds represent the total amount of funding that states have available to expand universal health coverage along three dimensions: 1) who is covered, and the proportion of population covered; 2) what services are covered; and 3) the extent to which the state covers the cost of these services. Under a right to health approach, this total level of funding will be derived from the maximum of available resources that states are required to dedicate to the right to health and other rights.

The human right to health, an international treaty obligation, provides critical insight into how states should work towards universal coverage (Figure 1). Core obligations offer benchmarks to assess progress towards universal coverage, such as non-discrimination, equitable distribution of health facilities, and essential services for all, including those addressing underlying determinants of health [13].

The core principle of equality requires states to prioritize covering 100% of their populations. Although 100% coverage of all health services will not be possible immediately, full coverage of “key” health interventions should be an initial benchmark towards universal coverage. The right to health framework militates against a narrow definition of “key” services. Rather, these should encompass WHO's health system building blocks (e.g., services, workforce, information, financing, and governance); essential vaccines, medicines, and technologies; and fundamental human needs (e.g., sanitation, nutritious food, potable water, safe housing, vector abatement, tobacco control, and healthy environments).

Critically, universal coverage should be re-conceptualized to encompass fundamental human needs given their major impact on health. Within this framework, specific services would be determined nationally through participatory processes [14].

The provision of each of these core entitlements—health systems, essential vaccines and medicines, and fundamental human needs—should represent only one significant step towards achieving the highest attainable standard of health. States, even wealthy ones, will need to continue to progress towards universal coverage. The right to health requires states to spend the “maximum of…available resources” towards progressively realizing health and other socioeconomic rights [5]. Thus, under international law, states have a duty “to move as expeditiously and effectively as possible towards” fully realizing the right to health [13].

*2. What responsibilities do all states have for the health of their own populations?*

The right to health places the primary responsibility on governments to ensure the health needs of all their inhabitants. National responsibility includes health sector funding, addressing the socioeconomic determinants of health, and good governance.

There is no universally agreed level of health sector funding adequate to meet the population's needs. African heads of state agreed to a benchmark of at least 15% of national budgets devoted to the health sector [15], and to allocating at least 10% of their national budgets for agricultural development [16]. Additionally, 32 African countries set a target, as an aspiration, to have public sector budget allocations for sanitation and hygiene programs reach at least 0.5% of gross domestic product [17].

These benchmarks set a minimum bar for national funding responsibilities, which extend beyond the health sector. National health responsibilities should comply with well-defined, measurable international standards, balanced against the flexibility necessary to respect national priorities, health profiles, and needs.

States also have a responsibility to govern well, derived from central human rights tenets such as participatory processes, transparent and accountable government, and non-discrimination and equality. Well-designed legal rules and institutional arrangements can facilitate honest administrations, openness, and accountability, along with meaningful civil society and community participation in decision-making. The law, moreover, should guarantee equality and non-discrimination on the basis of race, sex, religion, disability, and other statuses. Measures to enhance accountability to communities in India's National Rural Health Mission [18], and Brazilian policies to reduce health disparities [19], offer instructive lessons.

*3. What duties do states owe to people beyond their borders in securing the right to health?*

Resource-poor states lack capacity to ensure all of their people even core health goods and services, much less a fuller realization of the right to health. Countries in a position to assist are obliged to do so under principles of international law and global social justice. The Committee on Social, Economic and Cultural Rights has declared that cooperation towards realizing the right to health is “an obligation of all States,” particularly those “in a position to assist others” [13],[20]. All countries have mutual responsibilities towards ensuring the health of the world's most disadvantaged.

Beyond development assistance, coordination and coherence is required across sectors, as global health can be improved or harmed through state and international policies and rules that govern areas such as trade, intellectual property, health worker migration, international financing, and debt relief. These responsibilities extend to the exercise of state power and influence over multilateral institutions such as the World Bank, International Monetary Fund, and World Trade Organization.

International aspects of the right to health are ill-defined. With limited exceptions, such as the commitment of wealthy countries to spend 0.7% of gross national product on official development assistance, health and development commitments are framed collectively, vaguely, or not at all. Even when countries make commitments, they often fail to follow through. For example, only one month after countries at the 2010 United Nations (UN) MDG Summit committed to provide “adequate funding” for the Global Fund to Fight AIDS, Tuberculosis and Malaria, pledges at the replenishment conference fell billions of dollars short [21],[22]. The Summit called for accelerated development assistance for health, though the rate of increase in assistance dropped during the global recession [21],[23]. Budget shortfalls in the aftermath of the financial downturn further threaten assistance levels.

*4. What kind of global governance for health is needed to ensure that all states live up to their mutual responsibilities?*

Translating a shared understanding of national and global responsibilities into new realities requires effective and democratic global governance for health. Notwithstanding the Paris Declaration on Aid Effectiveness, global health faces challenges of weak leadership, poor coordination, underfunded priorities, and a lack of transparency, accountability, and enforcement [24].

Innovative global governance and enhanced funding would empower WHO to exercise effective leadership in the health sector and persuasive advocacy on agriculture, finance, and trade. Moreover, state policies (e.g., agricultural subsidies, intellectual property, and foreign affairs) can powerfully affect health in resource-poor countries. States, therefore, should adopt a “health-in-all-policies” approach where all ministries address the health impacts of their policies and programs. Effective governance must include active citizen participation to ensure transparency, collaboration, and accountability while maximizing creativity and resource mobilization by states, international organizations, businesses, and civil society.

Most importantly, the global health architecture must hold stakeholders accountable, with clear standards for success, monitoring progress, and enforcement—all of which have been lacking. Lack of sufficiently precise obligations and compliance mechanisms under the right to health hinders accountability, though promising approaches exist. Human rights bodies and UN special rapporteurs are adding clarity to state responsibilities under the right to health, which is required for meaningful accountability, as are constitutional court decisions in Argentina, India, and South Africa [25],[26].

Innovations in human rights law and practice hold potential for greater accountability. Regional right-to-health special rapporteurs could be established, enabling more effective national engagement. An empowered human rights sector could learn from international regimes with more vigorous adjudication and enforcement mechanisms, such as trade. Actions to ensure that social movements and voters are well-informed about their countries' commitments could strengthen political accountability.

The Global South, where most of the world's least healthy people reside, should lead in shaping global governance for health policies, where community priorities drive global action. New governance requires the full participation of, and support for, marginalized populations.

## Towards a Hopeful Future for Global Health

Why would states agree to greater accountability when so many countries fail to adhere to existing commitments? We do not underestimate the gravity of the challenge, yet JALI offers possibilities for success. Social mobilization could ignite new possibilities, as the AIDS movement has done, unleashing the collective power of health advocates and empowered communities.

The framework of mutual responsibilities that emerges from JALI should prove attractive to both Southern and Northern governments, creating incentives to develop a far-reaching global health agreement. Mutual responsibilities come with mutual benefits. Southern countries will benefit from increased respect for their strategies, greater and more predictable funding from more coordinated and accountable development partners, reform of policies that harm health, such as those in trade and agriculture, and most importantly, better health for their populations. Countries in the North will benefit from increased confidence that development assistance is spent effectively and the prospect of reduced financing needs over time as host countries increase their own health spending and build sustainable health systems. All will benefit from lessons on shared health challenges, from the economic and educational gains that will come with improved global health, and from increased protection for their populations from global public health threats—and from mutual goodwill derived from participating in an historic venture to make unprecedented progress towards global health equity.

This is also a moment of rare opportunity. The post-MDG global health framework is yet to be developed. Demand for accountability is growing. The right to health is increasingly motivating not only civil society, but also governments. The Pan American Health Organization passed a resolution on health and human rights [27], and the UN General Assembly explicitly recognized the right to clean water and sanitation [4]. Universal coverage, primary health care, and socioeconomic determinants are receiving renewed focus. Global health remains prominent on the international agenda, evidenced by the attention to global health and foreign policy and the upcoming UN high-level summit on non-communicable diseases.

In January 2011, WHO's Executive Board called on the Organization to assume a “more active and effective role” in “directing and coordinating” international health activities [28]. The agency initiated a reform process to strengthen its “central role in global health governance” [28],[29]. JALI supports WHO leadership, but also governance reforms extending beyond WHO, and even beyond the health sector, for a deeper understanding of the multiple forms of injustice that adversely affect health and development.

We invite readers to join JALI (http://www.section27.org.za/2010/11/23/jali) to develop widely shared understandings of national and global responsibilities for health to inform post-MDG commitments and create an innovative global agreement. It is time to define—and to meet—these responsibilities.

## Abbreviations

JALI: Joint Action and Learning Initiative on National and Global Responsibilities for Health
MDG: Millennium Development Goal
NGO: nongovernmental organization
UN: United Nations
WHO: World Health Organization
